# Crystal structure of 4-{[(1*H*-1,2,4-triazol-1-yl)meth­yl]sulfan­yl}phenol

**DOI:** 10.1107/S1600536814019965

**Published:** 2014-09-13

**Authors:** Yong-Le Zhang, Chuang Zhang, Wei Guo, Jing Wang

**Affiliations:** a School of Pharmaceutial Science, Hebei Medical University, Shijiazhuang 050017, People’s Republic of China

**Keywords:** crystal structure, 1,2,4-triazole derivative, hydrogen bonding, π–π stacking, biological activity

## Abstract

In the title compound, C_9_H_9_N_3_OS, the plane of the benzene ring forms a dihedral angle of 33.40 (5)° with that of the triazole group. In the crystal, mol­ecules are linked by O—H⋯N hydrogen bonds involving the phenol –OH group and one of the unsubstituted N atoms of the triazole ring, resulting in chains along [010]. These chains are further extended into a layer parallel to (001) by weak C—H⋯N hydrogen-bond inter­actions. Aromatic π–π stacking [centroid–centroid separation = 3.556 (1) Å] between the triazole rings links the layers into a three-dimensional network.

## Related literature   

For the biological activity of related compounds, see: Sidwell *et al.* (1972 ▶); Khan *et al.* (2010 ▶); Xu *et al.* (2011 ▶); Jubie *et al.* (2011 ▶); Patel *et al.* (2013 ▶); Salgın-Gökşen *et al.* (2007 ▶); Lin *et al.* (2005 ▶); Coucouvanis (2007 ▶). 
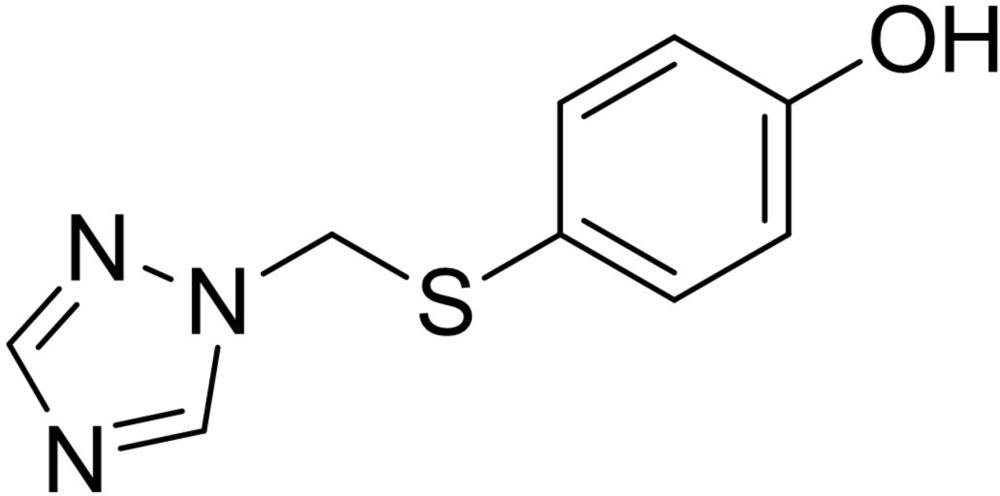


## Experimental   

### Crystal data  

C_9_H_9_N_3_OS
*M* *_r_* = 207.25
Monoclinic, 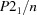

*a* = 5.3975 (10) Å
*b* = 10.0099 (19) Å
*c* = 18.311 (3) Å
β = 91.010 (3)°
*V* = 989.2 (3) Å^3^
*Z* = 4
Mo *K*α radiation
μ = 0.30 mm^−1^
*T* = 296 K
0.28 × 0.22 × 0.20 mm

### Data collection  

Bruker APEXII CCD area-detector diffractometer
Absorption correction: multi-scan (*SADABS*; Bruker, 2001 ▶) *T*_min_ = 0.922, *T*_max_ = 0.943
4977 measured reflections
1755 independent reflections
1453 reflections with *I* > 2σ(*I*)
*R*_int_ = 0.020

### Refinement  

*R*[*F* ^2^ > 2σ(*F* ^2^)] = 0.032
*wR*(*F* ^2^) = 0.077
*S* = 1.05
1755 reflections
128 parameters
H-atom parameters constrained
Δρ_max_ = 0.14 e Å^−3^
Δρ_min_ = −0.17 e Å^−3^

### 

Data collection: *APEX2* (Bruker, 2003 ▶); cell refinement: *APEX2*; data reduction: *SAINT* (Bruker, 2001 ▶); program(s) used to solve structure: *SHELXS97* (Sheldrick, 2008 ▶); program(s) used to refine structure: *SHELXL97* (Sheldrick, 2008 ▶); molecular graphics: *SHELXTL* (Sheldrick, 2008 ▶); software used to prepare material for publication: *SHELXTL*.

## Supplementary Material

Crystal structure: contains datablock(s) I. DOI: 10.1107/S1600536814019965/ds2243sup1.cif

Structure factors: contains datablock(s) I. DOI: 10.1107/S1600536814019965/ds2243Isup2.hkl

Supporting information file. DOI: 10.1107/S1600536814019965/ds2243Isup3.cml

**Fig. 1.**
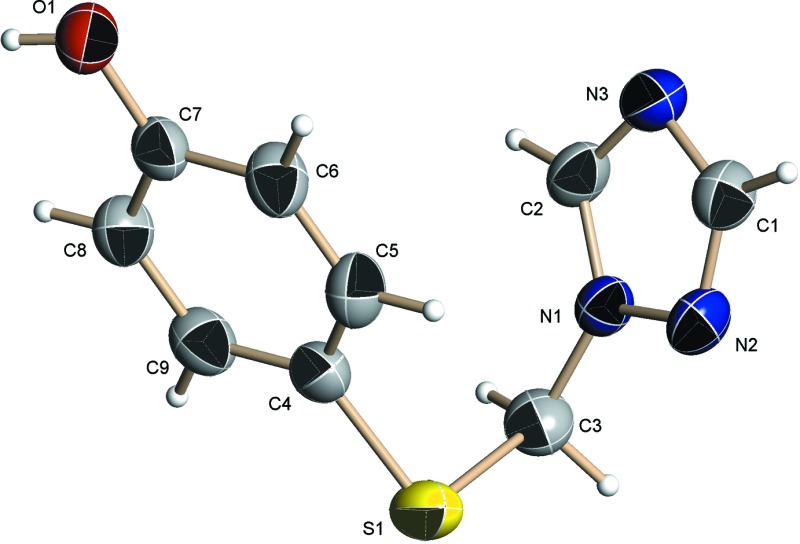
The molecular structure of the title compound with the atom numbering scheme. The displacement ellipsoids are drawn at the 40% probability level.

. DOI: 10.1107/S1600536814019965/ds2243fig1.tif
The mol­ecular structure of the title compound with the atom numbering scheme. The displacement ellipsoids are drawn at the 40% probability level.

**Fig. 2.**
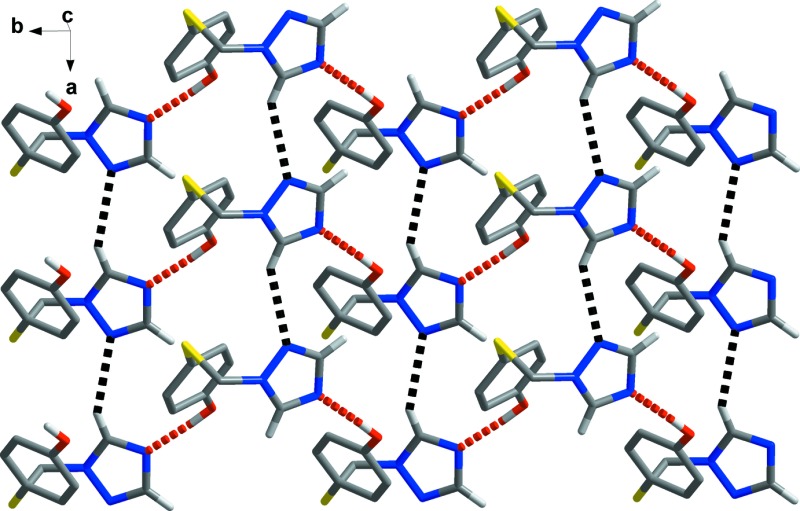
A view of the hydrogen bonded polymeric layer. The hydrogen bonds are shown as dashed lines.

. DOI: 10.1107/S1600536814019965/ds2243fig2.tif
A view of the hydrogen bonded polymeric layer. The hydrogen bonds are shown as dashed lines.

CCDC reference: 1022888

Additional supporting information:  crystallographic information; 3D view; checkCIF report

## Figures and Tables

**Table 1:** Hydrogen-bond geometry (Å, °)

*D*—H⋯*A*	*D*—H	H⋯*A*	*D*⋯*A*	*D*—H⋯*A*
O1—H1⋯N3^i^	0.82	1.91	2.7290 (19)	173
C2—H2⋯N2^ii^	0.93	2.55	3.318 (2)	140

Symmetry codes: (i) 
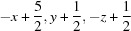
; (ii) 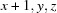.

## supplementary crystallographic information

### S1. Comment

1,2,4-triazole derivatives and sulfur-containing compounds have attracted much attention recently, owing to their fascinating and effective biological activities, for instance, anti­viral, anti­microbial, anti­cancer, analgesic, anti­oxidant as well as anti­inflammatory properties(Sidwell *et al.*,1972; Khan *et al.*, 2010; Xu *et al.*, 2011; Jubie *et al.*, 2011; Patel *et al.*, 2013; Salgın-Gökşen *et al.*, 2007; Lin *et al.*, 2005). As a result, much effort has been devoted to improve the activity of these compounds by modulating or introducing the substituents on the 1,2,4-triazole species. Among these, the thio­ether substituted 1,2,4-triazol ring systems represent an attractive group of substance that are promising for particular applications, such as bioinspired materials and biocatalysts (Coucouvanis, 2007). In this work, the title compound has been prepared and its crystal structure has been determined.

The crystal structure is illustrated in Fig. 1. Single crystal X-ray analysis reveals this compound crystallizes in monoclinic system with space group *P*2_1_/*n*. The bond lengths of C1—N2 [1.308 (2) Å] and C2—N3 [1.317 (2) Å] confirm they are double bonds. The dihedral angle formed by the benzene ring system and triazole plane is 33.396 (53)°, and the torsion angle of C4—S1—C3—N1 is 55.531 (131) Å.

Further analysis of crystal packing shows that these molecules are head-to-tail linked by O—H···N hydrogen bonds (Fig. 2, red dashed lines) between the phenolic hydroxyl groups and the triazole rings, forming a zigzag chain along the [010] axis. These one dimensional motifs are further extended to a two dimensional layer via weak C—H···N inter­actions (Fig. 2, black dashed lines). The layers arrange in an ABAB fashion along [001] direction and eventually constructed a three dimensional supra­molecular framework by virtue of π···π forces between the parallel triazole rings of neighbering molecules, with the centroid-to-centroid distance of 3.556 Å.

### S2. Experimental

1-chloro­methyl-1,2,4-triazole hydro­chloride (28 g, 0.18 mol), 4-mercaptophenol (22.7 g, 0.18 mol) and NaOH (24 g, 0.6 mol) were dissolved in 100 ml ethanol/water (v/v = 1/4) solution. The mixture was stirred at room temperature for 2 h, and further refluxed for 2 h longer. After the reaction was cooled to room temperature, 150 ml water was added to dissolve the generated precipitate. The mixture was acidified to pH 4 by dropwise addition of concd. hydro­chloric acid. The resulting voluminous white precipitate was filtered off, washed throughly with water and dried in air. The colorless strip crystals of the title compound were obtained by recrystallizing the powder samples from ethanol solution (yield 77%, m.p. 480-482 K).

### S3. Refinement

H atoms bonded to C were positioned with idealized geometry using a riding model with the aromatic C—H = 0.93 Å and methyl­ene C—H = 0.97 Å, respectively. The H atom attached to O atom was found in difference electron-density maps and fixed O—H = 0.82 Å bond length. All H atoms were refined with isotropic displacement parameters set at *U*_iso_(H) = 1.2 *U*_eq_(C) and *U*_iso_(H) =1.5 *U*_eq_(O) of the parent atom.

### Crystal data

**Table:**

C_9_H_9_N_3_OS	*F*(000) = 432
*M**_r_* = 207.25	*D*_x_ = 1.392 Mg m^−^^3^
Monoclinic, *P*2_1_/*n*	Mo *K*α radiation, λ = 0.71073 Å
Hall symbol: -P 2yn	Cell parameters from 1691 reflections
*a* = 5.3975 (10) Å	θ = 2.3–23.8°
*b* = 10.0099 (19) Å	µ = 0.30 mm^−^^1^
*c* = 18.311 (3) Å	*T* = 296 K
β = 91.010 (3)°	Strip, colorless
*V* = 989.2 (3) Å^3^	0.28 × 0.22 × 0.20 mm
*Z* = 4	

### Data collection

**Table:**

Bruker APEXII CCD area-detector diffractometer	1755 independent reflections
Radiation source: fine-focus sealed tube	1453 reflections with *I* > 2σ(*I*)
Graphite monochromator	*R*_int_ = 0.020
phi and ω scans	θ_max_ = 25.0°, θ_min_ = 2.2°
Absorption correction: multi-scan (*SADABS*; Bruker, 2001)	*h* = −6→6
*T*_min_ = 0.922, *T*_max_ = 0.943	*k* = −11→11
4977 measured reflections	*l* = −18→21

### Refinement

**Table:**

Refinement on *F*^2^	Primary atom site location: structure-invariant direct methods
Least-squares matrix: full	Secondary atom site location: difference Fourier map
*R*[*F*^2^ > 2σ(*F*^2^)] = 0.032	Hydrogen site location: inferred from neighbouring sites
*wR*(*F*^2^) = 0.077	H-atom parameters constrained
*S* = 1.05	*w* = 1/[σ^2^(*F*_o_^2^) + (0.0294*P*)^2^ + 0.2664*P*] where *P* = (*F*_o_^2^ + 2*F*_c_^2^)/3
1755 reflections	(Δ/σ)_max_ < 0.001
128 parameters	Δρ_max_ = 0.14 e Å^−^^3^
0 restraints	Δρ_min_ = −0.17 e Å^−^^3^

### Special details

**Table:**

Geometry. All e.s.d.'s (except the e.s.d. in the dihedral angle between two l.s. planes) are estimated using the full covariance matrix. The cell e.s.d.'s are taken into account individually in the estimation of e.s.d.'s in distances, angles and torsion angles; correlations between e.s.d.'s in cell parameters are only used when they are defined by crystal symmetry. An approximate (isotropic) treatment of cell e.s.d.'s is used for estimating e.s.d.'s involving l.s. planes.
Refinement. Refinement of *F*^2^ against ALL reflections. The weighted *R*-factor *wR* and goodness of fit *S* are based on *F*^2^, conventional *R*-factors *R* are based on *F*, with *F* set to zero for negative *F*^2^. The threshold expression of *F*^2^ > σ(*F*^2^) is used only for calculating *R*-factors(gt) *etc*. and is not relevant to the choice of reflections for refinement. *R*-factors based on *F*^2^ are statistically about twice as large as those based on *F*, and *R*- factors based on ALL data will be even larger.

### Fractional atomic coordinates and isotropic or equivalent isotropic displacement parameters (Å^2^)

**Table:**

	*x*	*y*	*z*	*U*_iso_*/*U*_eq_	
S1	0.66005 (10)	0.44273 (5)	0.08502 (3)	0.05715 (18)	
O1	1.2294 (3)	0.31388 (12)	0.35509 (7)	0.0530 (4)	
H1	1.3233	0.3747	0.3671	0.080*	
N1	0.8498 (2)	0.20284 (13)	0.04543 (7)	0.0369 (3)	
N2	0.6462 (2)	0.12317 (15)	0.04545 (8)	0.0450 (4)	
N3	0.9755 (3)	0.01570 (15)	0.09366 (8)	0.0475 (4)	
C1	0.7326 (3)	0.01291 (19)	0.07461 (9)	0.0461 (4)	
H1A	0.6339	−0.0620	0.0817	0.055*	
C2	1.0418 (3)	0.13729 (18)	0.07465 (9)	0.0440 (4)	
H2	1.2004	0.1724	0.0808	0.053*	
C3	0.8344 (3)	0.34022 (18)	0.02238 (10)	0.0483 (5)	
H3A	0.7561	0.3442	−0.0257	0.058*	
H3B	1.0006	0.3762	0.0185	0.058*	
C4	0.8297 (3)	0.40921 (17)	0.16692 (9)	0.0436 (4)	
C5	0.7646 (3)	0.30194 (19)	0.21040 (10)	0.0487 (5)	
H5	0.6281	0.2499	0.1974	0.058*	
C6	0.9001 (4)	0.27158 (18)	0.27266 (10)	0.0487 (5)	
H6	0.8549	0.1990	0.3012	0.058*	
C7	1.1036 (3)	0.34846 (16)	0.29307 (9)	0.0403 (4)	
C8	1.1682 (3)	0.45690 (18)	0.25025 (10)	0.0458 (4)	
H8	1.3035	0.5096	0.2636	0.055*	
C9	1.0315 (4)	0.48657 (18)	0.18780 (10)	0.0477 (5)	
H9	1.0757	0.5595	0.1594	0.057*	

### Atomic displacement parameters (Å^2^)

**Table:**

	*U*^11^	*U*^22^	*U*^33^	*U*^12^	*U*^13^	*U*^23^
S1	0.0629 (3)	0.0550 (3)	0.0531 (3)	0.0208 (2)	−0.0116 (2)	−0.0047 (2)
O1	0.0666 (9)	0.0444 (7)	0.0476 (7)	−0.0068 (6)	−0.0120 (6)	0.0013 (6)
N1	0.0317 (7)	0.0419 (8)	0.0370 (8)	−0.0003 (6)	−0.0013 (6)	−0.0019 (6)
N2	0.0338 (8)	0.0530 (9)	0.0481 (9)	−0.0058 (7)	−0.0062 (6)	−0.0003 (7)
N3	0.0478 (9)	0.0500 (9)	0.0443 (9)	0.0054 (7)	−0.0067 (7)	0.0001 (7)
C1	0.0470 (11)	0.0484 (11)	0.0427 (10)	−0.0062 (8)	−0.0011 (8)	0.0010 (8)
C2	0.0309 (9)	0.0542 (12)	0.0466 (10)	0.0011 (8)	−0.0041 (7)	−0.0057 (9)
C3	0.0553 (11)	0.0473 (11)	0.0424 (10)	0.0003 (9)	−0.0001 (8)	0.0043 (8)
C4	0.0471 (10)	0.0402 (10)	0.0434 (10)	0.0082 (8)	−0.0015 (8)	−0.0072 (8)
C5	0.0476 (10)	0.0489 (11)	0.0496 (11)	−0.0088 (8)	−0.0010 (8)	−0.0080 (9)
C6	0.0609 (12)	0.0402 (10)	0.0449 (10)	−0.0115 (9)	0.0023 (9)	0.0005 (8)
C7	0.0473 (10)	0.0369 (9)	0.0366 (9)	0.0017 (8)	0.0012 (7)	−0.0057 (7)
C8	0.0463 (10)	0.0414 (10)	0.0495 (11)	−0.0077 (8)	−0.0002 (8)	−0.0027 (8)
C9	0.0583 (11)	0.0376 (10)	0.0473 (11)	−0.0001 (8)	0.0044 (9)	0.0021 (8)

### Geometric parameters (Å, º)

**Table:**

S1—C4	1.7753 (18)	C3—H3A	0.9700
S1—C3	1.8146 (18)	C3—H3B	0.9700
O1—C7	1.358 (2)	C4—C9	1.385 (3)
O1—H1	0.8200	C4—C5	1.386 (3)
N1—C2	1.331 (2)	C5—C6	1.378 (3)
N1—N2	1.3578 (18)	C5—H5	0.9300
N1—C3	1.440 (2)	C6—C7	1.387 (2)
N2—C1	1.308 (2)	C6—H6	0.9300
N3—C2	1.317 (2)	C7—C8	1.387 (2)
N3—C1	1.351 (2)	C8—C9	1.382 (2)
C1—H1A	0.9300	C8—H8	0.9300
C2—H2	0.9300	C9—H9	0.9300
			
C4—S1—C3	99.29 (8)	C9—C4—C5	118.74 (17)
C7—O1—H1	109.5	C9—C4—S1	121.27 (14)
C2—N1—N2	109.56 (14)	C5—C4—S1	119.97 (14)
C2—N1—C3	128.98 (15)	C6—C5—C4	120.68 (17)
N2—N1—C3	121.22 (14)	C6—C5—H5	119.7
C1—N2—N1	102.31 (13)	C4—C5—H5	119.7
C2—N3—C1	102.59 (15)	C5—C6—C7	120.46 (17)
N2—C1—N3	115.15 (16)	C5—C6—H6	119.8
N2—C1—H1A	122.4	C7—C6—H6	119.8
N3—C1—H1A	122.4	O1—C7—C6	117.77 (15)
N3—C2—N1	110.40 (15)	O1—C7—C8	123.04 (15)
N3—C2—H2	124.8	C6—C7—C8	119.19 (16)
N1—C2—H2	124.8	C9—C8—C7	120.00 (17)
N1—C3—S1	112.49 (12)	C9—C8—H8	120.0
N1—C3—H3A	109.1	C7—C8—H8	120.0
S1—C3—H3A	109.1	C8—C9—C4	120.93 (17)
N1—C3—H3B	109.1	C8—C9—H9	119.5
S1—C3—H3B	109.1	C4—C9—H9	119.5
H3A—C3—H3B	107.8		
			
C2—N1—N2—C1	−0.58 (18)	C3—S1—C4—C5	−88.92 (16)
C3—N1—N2—C1	−175.34 (14)	C9—C4—C5—C6	−0.9 (3)
N1—N2—C1—N3	0.3 (2)	S1—C4—C5—C6	177.54 (14)
C2—N3—C1—N2	0.1 (2)	C4—C5—C6—C7	0.3 (3)
C1—N3—C2—N1	−0.45 (19)	C5—C6—C7—O1	179.81 (16)
N2—N1—C2—N3	0.68 (19)	C5—C6—C7—C8	0.4 (3)
C3—N1—C2—N3	174.91 (15)	O1—C7—C8—C9	−179.87 (16)
C2—N1—C3—S1	−105.58 (18)	C6—C7—C8—C9	−0.5 (3)
N2—N1—C3—S1	68.07 (18)	C7—C8—C9—C4	−0.1 (3)
C4—S1—C3—N1	55.54 (14)	C5—C4—C9—C8	0.8 (3)
C3—S1—C4—C9	89.51 (16)	S1—C4—C9—C8	−177.62 (13)

### Hydrogen-bond geometry (Å, º)

**Table:**

*D*—H···*A*	*D*—H	H···*A*	*D*···*A*	*D*—H···*A*
O1—H1···N3^i^	0.82	1.91	2.7290 (19)	173
C2—H2···N2^ii^	0.93	2.55	3.318 (2)	140

Symmetry codes: (i) −*x*+5/2, *y*+1/2, −*z*+1/2; (ii) *x*+1, *y*, *z*.
