# Supplementary figures and images for: Crystal structure of 4-{[(1H-1,2,4-triazol-1-yl)meth­yl]sulfan­yl}phenol
Source: Acta Crystallogr Sect E Struct Rep Online. 2014 Sep 13;70(Pt 10):o1106. doi: 10.1107/S1600536814019965 (PMC4257201; doi:10.1107/S1600536814019965)

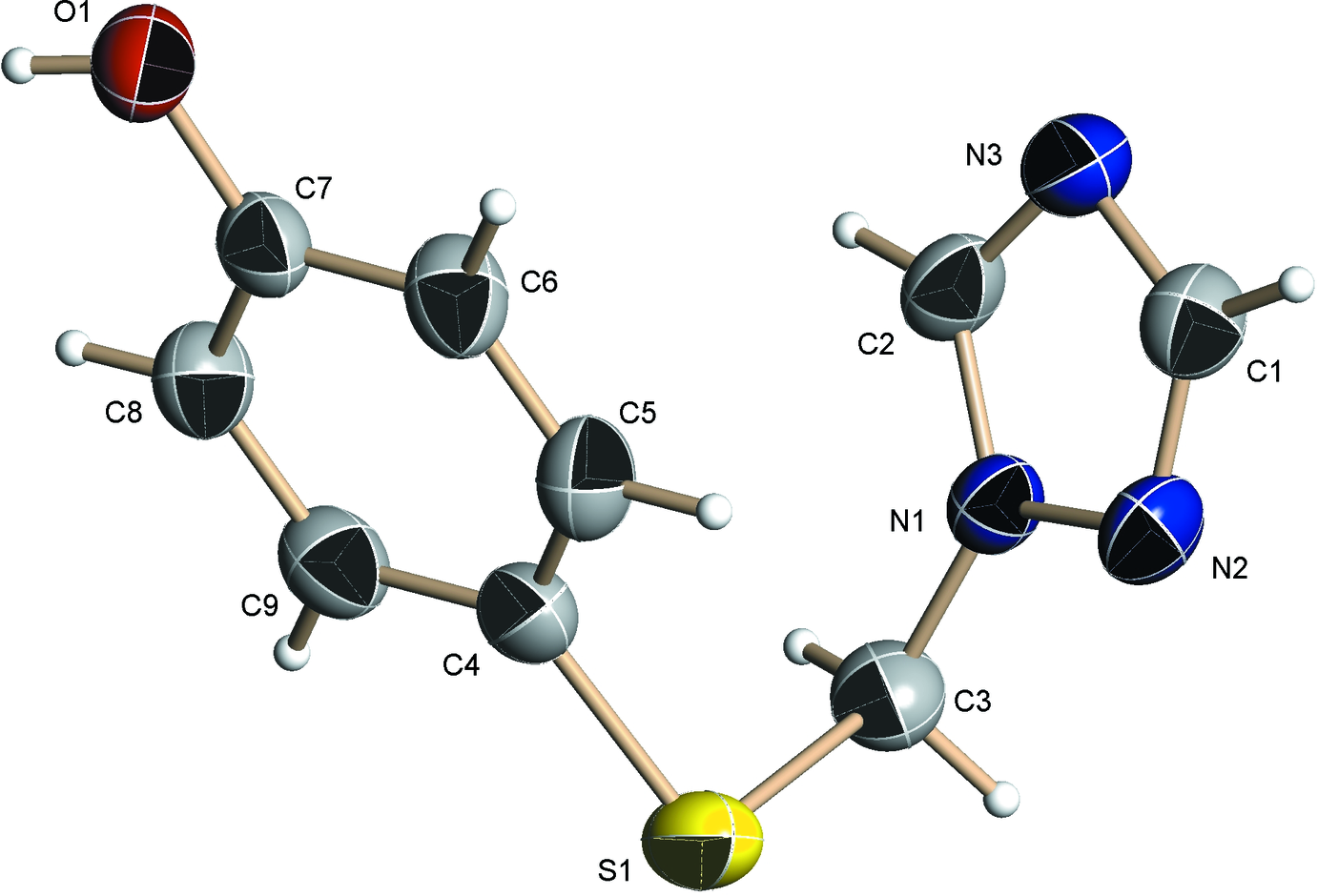

Supplement: Supplementary file 4 [file e-70-o1106-fig1.tif]

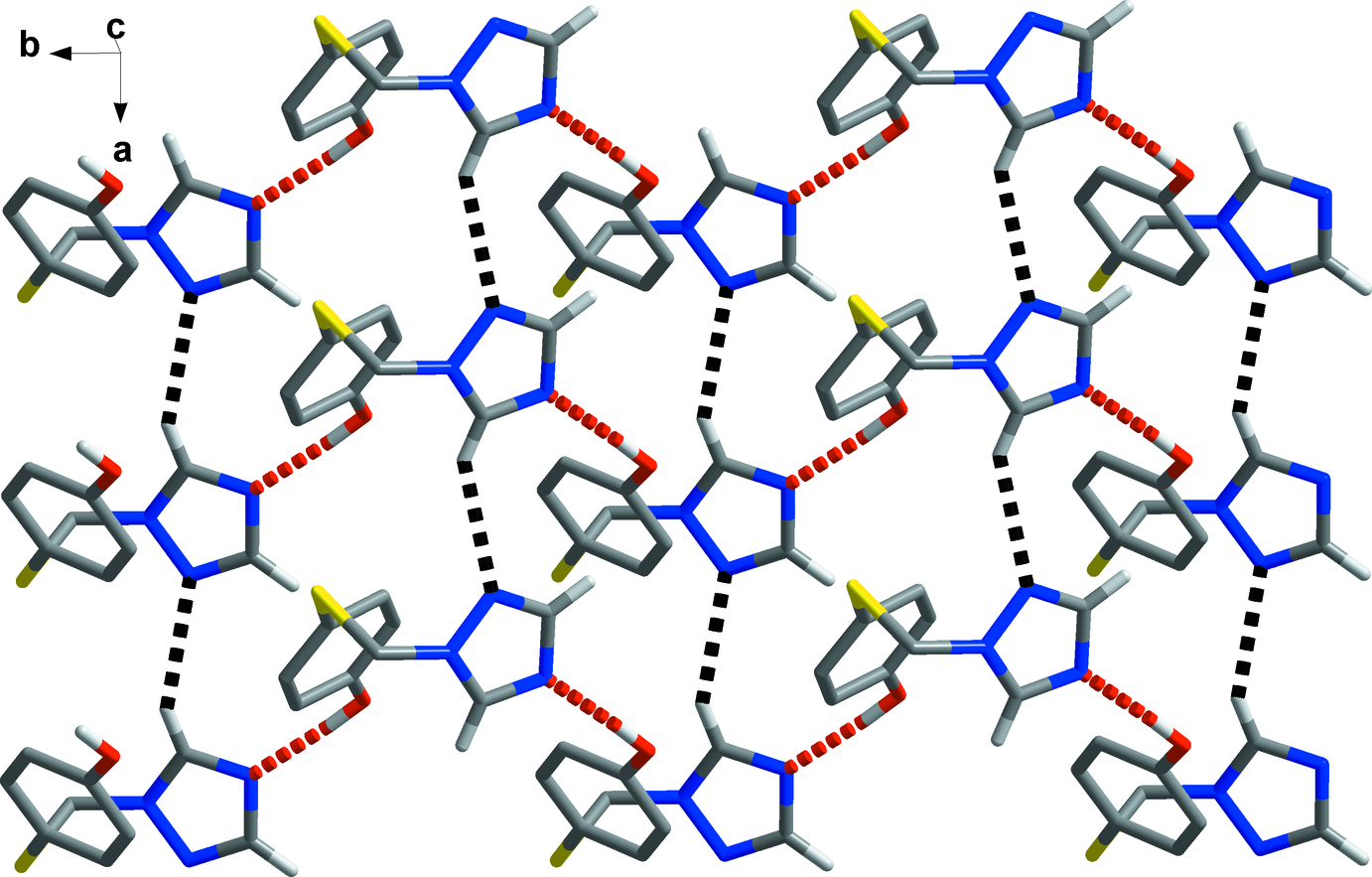

Supplement: Supplementary file 5 [file e-70-o1106-fig2.tif]
